# Impact of stress, fear and anxiety on the nociceptive responses of larval zebrafish

**DOI:** 10.1371/journal.pone.0181010

**Published:** 2017-08-02

**Authors:** Javier Lopez-Luna, Qussay Al-Jubouri, Waleed Al-Nuaimy, Lynne U. Sneddon

**Affiliations:** 1 Department of Evolution, Ecology and Behaviour, Institute of Integrative Biology, University of Liverpool. Liverpool, United Kingdom; 2 Department of Electrical Engineering and Electronics, University of Liverpool, Liverpool, United Kingdom; Chinese Academy of Sciences, CHINA

## Abstract

Both adult and larval zebrafish have been demonstrated to show behavioural responses to noxious stimulation but also to potentially stress- and fear or anxiety- eliciting situations. The pain or nociceptive response can be altered and modulated by these situations in adult fish through a mechanism called stress-induced analgesia. However, this phenomenon has not been described in larval fish yet. Therefore, this study explores the behavioural changes in larval zebrafish after noxious stimulation and exposure to challenges that can trigger a stress, fear or anxiety reaction. Five-day post fertilization zebrafish were exposed to either a stressor (air emersion), a predatory fear cue (alarm substance) or an anxiogenic (caffeine) alone or prior to immersion in acetic acid 0.1%. Pre- and post-stimulation behaviour (swimming velocity and time spent active) was recorded using a novel tracking software in 25 fish at once. Results show that larvae reduced both velocity and activity after exposure to the air emersion and alarm substance challenges and that these changes were attenuated using etomidate and diazepam, respectively. Exposure to acetic acid decreased velocity and activity as well, whereas air emersion and alarm substance inhibited these responses, showing no differences between pre- and post-stimulation. Therefore, we hypothesize that an antinociceptive mechanism, activated by stress and/or fear, occur in 5dpf zebrafish, which could have prevented the larvae to display the characteristic responses to pain.

## Introduction

Nociception is the sensory mechanism used to perceive actual or potential tissue damage. The neurons that mediate nociceptive information are called nociceptors. Fish can perceive and respond to a wide range of stimuli, including mechanical [1], thermal [2], electrical [3] or chemical [4]. Recent investigations have reported the presence of these neurons in fish [1,5], which, along with the findings of nociceptive pathways and brain activity [6,7] and the regulation of novel candidate genes after a nociceptive event [8], suggest that fish are capable of nociception. Moreover, changes in the behaviour have been recorded after exposure to noxious stimulation [9–11] and these have been ameliorated by administering analgesia [5].

Pain can be considered as an evolutionarily developed defence response to an aversive or noxious stimulus [12]. Administration of potentially painful stimuli results is a wide range of physiological (changes in the heart rate, body temperature or respiratory rate) and behavioural responses (abnormal behaviours, reduction of activity, appetite) in mammals, birds, amphibians or fish [13–15]. The behavioural responses to a noxious stimulus can be modulated by an imposed restraint such as environmental stressors or concurrent experiences such as exposure to a predator [16]. This phenomenon is called stress-induced analgesia and refers to a reduced pain response after stress exposure, which is mediated by descending pain-inhibitory circuits and may be an indicator of adequate centrally mediated pain control [17]. In mammals, the endogenous antinociceptive system is a component of defensive behavior and can be mobilized in stress situations or during encounters where there is a risk of injury to the animal [12]. Indeed, acute exposure to stressors induce temporary analgesia in rats [18]. The existence of an endogenous antinociceptive system has been previously demonstrated in fish [19,20] and a study has provided evidence of the descending modulation of nociception in zebrafish [21]. Stress can be a powerful inhibitor of the nociceptive response in fish, as it affects the defensive strategies and the anti-predatory behaviour [22]. Ashley et al. [19] proved that social subordination elicited a higher antinociceptive response in rainbow trout and evidence was recently provided for the existence of an endogenous modulation of nociception in piaçu [23].

Antinociception associated with fearful experiences involving a confrontation with a predator or a predator being in close proximity is well studied in mammals [22], but very little is known about how fear affects antinociception responses in fish. The alarm substance, which is released by the injured skin of fish and that is known to elicit changes in the physiology and behaviour, warns conspecifics about predator activity [24]. Fear-like responses have been recorded after exposure to the alarm substance stimuli, [25,26] and in a recent study, antinociceptive-like behaviour was reduced in zebrafish after an alarm-induced reaction [21]. Similarly, anxiety can also modulate the nociceptive response in humans [27], other mammals [28] and fish [21]. Caffeine has been reported to increase anxiety-like behaviours in both adult [29] and larval zebrafish [30,31]. It is considered a stimulant at low doses but higher doses seem to increase general activity [32]. Both fear and anxiety are very closely related and sometimes undistinguishable. While fear is defined as a motivational state elicited by stimuli that give rise to a defensive behaviour or escape, anxiety is considered as a response to a threat or internal conflict [33]. Fear is generally thought to inhibit the pain response, whereas anxiety seems to enhance pain in humans [27,34]. In fish, there is little evidence of how these two behaviours regulate the nociceptive threshold [20] and only one study has explored the modulation of nociception by environmental stressors in zebrafish [21]. Previous research in our laboratory has reported altered activity after noxious chemical and thermal stimulation in 5dpf larval zebrafish that are reduced by lidocaine which has analgesic properties in fish [35,36] and other authors found similar evidence of behavioral changes in larvae undergoing painful challenges [11,37]. However, no studies have demonstrated whether these mechanisms of modulation of the nociceptive responses present in adult fish exist in the larval stages.

Under European legislation for experimental animals, fish are only protected when able to feed freely and in zebrafish this is six days post fertilization thus younger zebrafish are not covered by the legislation and can be considered as replacement for the use of adult fish. The objective of this study was to evaluate the behavioural responses of non-protected 5dpf zebrafish to stressful, fearful and anxiety-eliciting situations during noxious stimulation and thus validate their use as a replacement for adult forms. To determine the impact of stress larvae were exposed to air emersion, a standard stressor, and then exposed to a noxious chemical. For fear, larvae were exposed to conspecific alarm substance prior to the noxious event and for anxiety, caffeine was administered via immersion prior to noxious exposure. To confirm that these responses were mediated by stress, fear and anxiety, drugs that block the physiological stress response (specifically cortisol production, etomidate) and reduce anxiety and fear (diazepam) were employed. We showed that stress and fear or anxiety resulted in stress induced analgesia and reduced the responses to noxious treatment. These effects seem to be modulated by etomidate via a block on the stress response or via the anti-anxiety effects of diazepam.

## Materials and methods

### Experimental animals

All experiments were conducted according to the guidelines of research ethics as approved by the Ethics Committee at the University of Liverpool (40/3534). Five days post-fertilisation (dpf) zebrafish larvae of AB wild type were used for the purposes of this experiment. Eggs were provided by the in-house breeding programme. Adult zebrafish were held in breeding pairs and eggs collected the day after. Eggs were then kept in 3L plastic tanks (Pentair Aquatic Habitat, Apopka, USA) in a closed aerated recirculation system supplied with filtered, aerated freshwater at a temperature of 28.5 ±0.5°C and on a 12 h: 12 h light:dark cycle until 5 dpf. These were then selected for experiments. Water quality parameters were kept ideal for this species (pH 7.2; Nitrite = 0 ppm; Nitrate <20 ppm; Ammonia = 0 ppm). Any animals not used in the present study were either held as stock for other experiments or were humanely killed before reaching 6dpf by being placed in an Eppendorf on dry ice for use in another study investigating genomics.

### Apparatus

Larvae movements were analysed by placing them individually to eliminate group interactions in a custom-built plastic plate of 25 wells (length: 16.5mm; width: 16.5mm; depth: 8mm) with a 53μm mesh bottom (Zebrafish Management Ltd., UK), which allows water to be flushed in and out. The plate was positioned above an infrared light (illumination area 450 x 210 mm; 850 nm, Loligo Systems, Denmark) to facilitate the tracking of the fish. The experimental tank was supplied from a glass sump tank (45 x 35 x 40cm) with filtered and maintained at a constant temperature of 28.5±0.5°C and with aeration provided via an air stone (12cm) and air line from a compressed air supply.

Video of spontaneous free-swimming was recorded at 25 fp/s. In summary, behaviour of each larvae tracked using a digital monochrome infrared-sensitive camera (IDS UI-1240LE-NIR-GL; STEMMER IMAGING, Surrey, UK) with an attached lens (SPACE-COM JHF25M-5MP; SPACE inc., Tokyo, Japan) mounted above the plate. Videos were acquired without compression, via IDS software (uEye Cockpit; IDS Imaging Development Systems GmbH) via connection to a laptop computer (HP, DSC HM87, Palo Alto, CA, USA). For video analysis, a novel tracking software based on an object automated detection, tracking and monitoring algorithm was developed for this project. Data files generated by the tracking software were then processed with a bespoke algorithm in MATLAB, which can detect various behavioural larvae patterns larvae based upon standard motion features including average velocity (mm/s) and time spent active (%). Previous studies have demonstrated that these two parameters are affected by noxious, potentially painful stimuli in larval zebrafish [35,36].

### Experimental procedure

Testing occurred between 09:00 and 16:00 using a randomized trial design to eliminate systematic effects due to time of day. In all experiments, larvae were caught at random and gently pipetted from the rearing tank and placed in the individual wells (25 well plate) to acquire video recordings. Animals were then allowed 30 minutes to acclimate to the experimental arena. All groups of larvae were recorded for 10 minutes to assess the pre-stimulation behaviour (video recording 1) and recorded again at the end of the experimental procedures for another 10 minutes to assess the post-stimulation behaviour (video recording 2). For each of the treatments described below (see Table 1), 10 groups of 25 larvae per group were used (n = 10 per treatment).

**Table 1:** List of abbreviations and concentrations of the different substances used to assess the effect of air immersion, alarm pheromone, caffeine, etomidate, diazepam dissolved in DMSO and DMSO alone on the nociceptive-like response of 5 dpf (days post-fertilisation) zebrafish.

Group name	Substances	Group name	Substances
**CO/AE**	Control air emersion	**CO/AP**	Control alarm pheromone
**AC**	Acetic acid	**AP**	Alarm pheromone
**AC+LI**	Acetic acid	**AP+DI**	Alarm pheromone
	Lidocaine		Diazepam 5 mg l^-1^
**ET**	Etomidate	**AP+AC**	Alarm pheromone
			Acetic acid
**AE**	Air emersion	**CO/CF**	Control caffeine
**AE+ET**	Air emersion	**CF**	Caffeine
	Etomidate		
**AE+AC**	Air emersion	**CF+DI**	Caffeine
	Acetic acid		Diazepam
**DI**	Diazepam	**CF+AC**	Caffeine
			Acetic acid
**DMSO**	DMSO		

**Fig 1:**
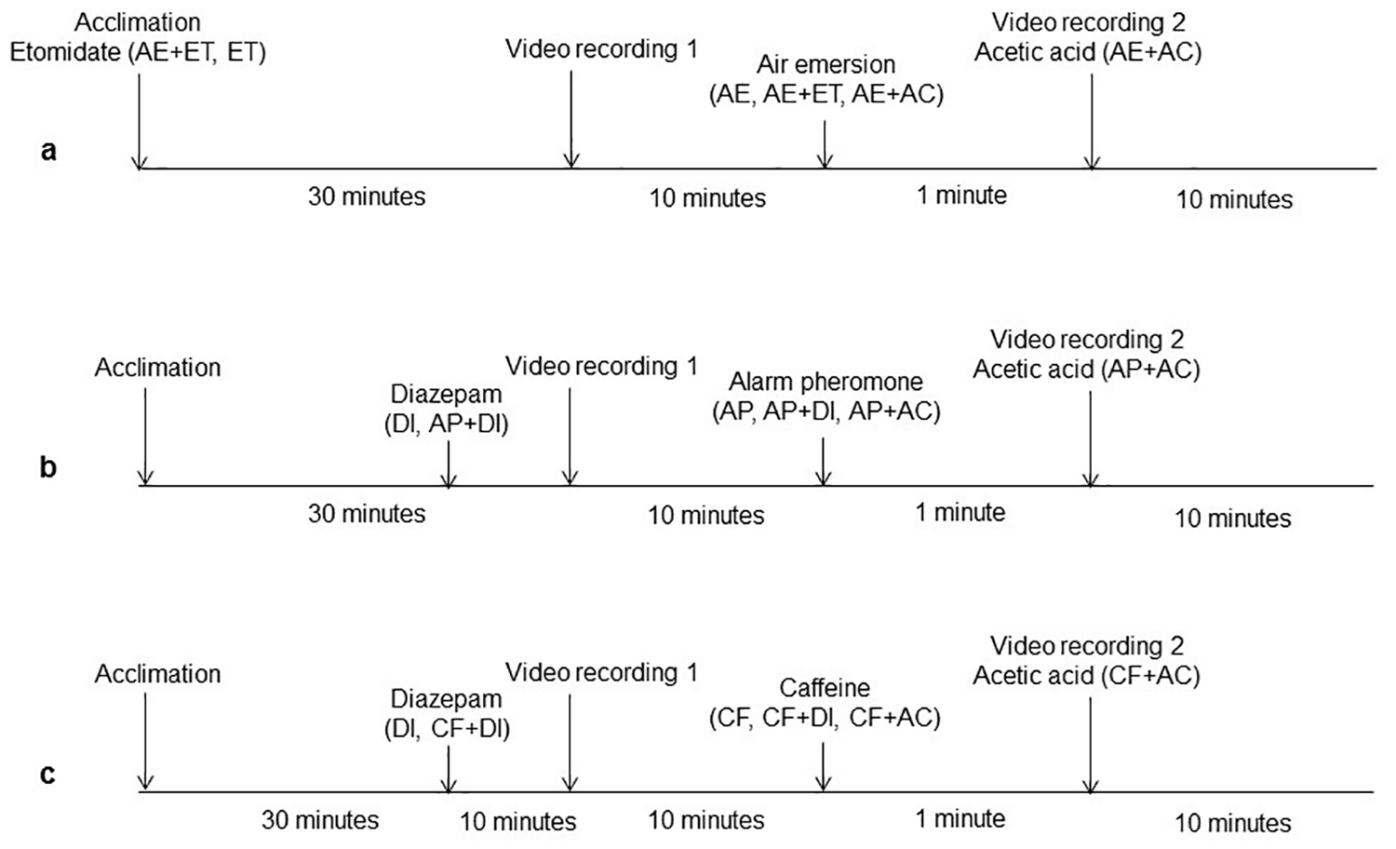
Diagram of the experimental procedures and group names (see Table 1). Assessment of the impact of air emersion (a), alarm pheromone (substance) (b) and caffeine (c) on the nociceptive-like responses of larval zebrafish. Video recording 1 and 2 indicate the pre- and post-stimulation video recordings, respectively. The names indicate the moment when the challenges were applied or when the substances were introduced in the tank water so only those groups in brackets received that particular treatment.

To test that the acetic acid is actually evoking a nociceptive-like response, a group of fish (group AC) was exposed to acetic acid 0.1% by adding the acid (APC Pure, Manchester, UK) to the tank water using a syringe right after video recording 1. This concentration was shown to be effective in previous studies [35,36]. Pilot experiments using food dye without larval presence showed this approached allowed complete mixing in the tank. Another group of fish (group AC+LI) followed the same procedure but dissolving a drug with analgesic properties, lidocaine (lidocaine hydrochloride monohydrate, Sigma-Aldrich Co., UK), in the tank water to a final concentration of 5 mg l^-1^ right before the fish were placed in the apparatus. All experimental procedures are shown in Fig 1.

To determine to what extent stress modulates the response to pain in larvae, a series of experiments were carried out. A first group of fish was exposed to a standard stressor, air emersion, for 1 minute by adjusting the water level in the experimental tank (group AE) right after video recording 1. The effect of an anaesthetic agent known to inhibit production of cortisol in fish and suppress physiological stress, etomidate [38], was tested in a second group of larvae (group AE+ET) by dissolving 0.5 mg l^-1^ of etomidate (Ark Pharm Inc., Libertyville, IL, USA) in the tank water immediately before the larvae were placed in the apparatus and then exposed to air emersion after video recording 1. The sole effect of etomidate 0.5 mg l^-1^ was tested in a third group of fish (group ET), following the same procedure above but without the air emersion challenge. Finally, a group of larvae (group AE+AC) were subjected to the air emersion challenge and then exposed to acetic acid 0.1% right before video recording 2. To determine the effect of any potential handling stress, a control group, which underwent the same experimental procedure as the AE group but left undisturbed, was included (group CO/AE).

To determine the impact of the alarm pheromone or alarm substance, a predator cue, on the pain response of larvae, a first group of fish (group AP) was exposed for 10 minutes to a concentration of 3.5 ml l^-1^ of alarm substance produced as proposed by Maximino [21] and then fish were video recorded (video recording 2). The effect of exposure for 10 minutes to an anxiolytic substance (diazepam in 0.005% dimethyl sulfoxide (DMSO)) on the alarm pheromone reaction was tested in a third group of larvae (AP+DI) by dissolving 5mg l^-1^ of the substance in the tank immediately before video recording 1 and then exposed to the alarm pheromone and recorded again for 10 minutes (video recording 2). The sole effect of diazepam 5 mg l^-1^ was tested in a group of animals (DI), following the same procedure above but without the alarm pheromone. Another group of larvae (AP+AC) was exposed for 10 minutes to a concentration of 3.5 ml l^-1^ of alarm pheromone and the appropriate amount of acetic acid 0.1% was subsequently added to the tank water. Finally, to determine the effect of any potential handling stress, a control group, which underwent the same experimental procedure as the group AP but left undisturbed, was included (CO/AP). An additional control consisting of 0.005% (v/v) DMSO in tank water was also used to rule out any undesirable effects of DMSO, following the same general procedure but adding the appropriate amount of DMSO to the tank water right before video recording 2.

The stimulatory effect of caffeine on the pain response of larval zebrafish was determined with a set of experiments: the first group of fish was exposed to 100 mg l^-1^ of caffeine (caffeine powder C0750, Sigma Aldrich Co, UK) for 10 minutes and then immediately recorded (video recording 2). The effect of diazepam (in 0.005% DMSO) on the caffeine reaction was tested in a third group of larvae (CF+DI) by dissolving 5 mg l^-1^ of this substance in the tank water 10 minutes prior to video recording 1 and then exposed to caffeine for 10 minutes and recorded again for 10 minutes (video recording 2). The sole effect of diazepam 5 mg l^-1^ was determined in a group of animals (DI), following the same procedure as above but without the addition of caffeine. Anxiolytic effects of diazepam are known to occur within 10 min of exposure in zebrafish [39,40].

Another group of fish (CF+AC) was exposed to 100 mg l^-1^ of caffeine for 10 minutes [30,31] and then acetic acid was added to the tank water for a final concentration of 0.1%. Fish were immediately recorded again for 10 minutes to assess the post-stimulation behaviour (video recording 2). Finally, a control group (CO/CF), which underwent the same experimental procedure as the CF group but left undisturbed, was included. The drugs and concentrations used in these experiments are listed in Table 2.

**Table 2:** List of drugs used in the experiment, concentrations and potential effects on zebrafish larvae. Concentrations were taken from published sources [21,29,31,40–42].

Substance	Concentration	Potential effect
Acetic acid	0.1%	Noxious
Lidocaine	5 mg l^-1^	Analgesic
Etomidate	0.5 mg l^-1^	Cortisol release blocker
Diazepam	5 mg l^-1^	Anxiolytic
DMSO	0.005%	-
Alarm pheromone	3.5 mg l^-1^	Predatory fear cue
Caffeine	100 mg l^-1^	Anxiogenic

### Statistical analysis

Statistical analyses were performed using SPSS version 22.0.0.1 software. Behavioural data, namely average velocity (mm/s), average acceleration (mm/s^2^), time active (%) and total distance moved (mm) did not fulfil the requirements of a normally distributed population (Kolmogorov-Smirnov; P<0.001) and of the homogeneity of variance (Levene’s test, P<0.001), however initial data analysis showed that only the velocity (mm/s) and the time spent active (%) were affected by noxious treatment. Therefore, for the purposes of this study, only the average velocity (mm/s) and the time spent active (%) were used as indicators of the larval behaviour. Observations where larvae showed no movements at all both before and after stimulation and those in which the tracking process could not successfully track both before and after exposure were excluded from the data set (13 observations per treatment on average, approximately 5.3%). Any significant variation in the behavioural response (velocity, mm/s and time active, %) of the fish during the day that could mask the impact of the different treatments used was determined. Observations in the control treatment in the three experiments were sorted into six groups depending on the time of the day when they were taken (09h00, 10h00, 11h00, 12h00, 13h00 and 14h00) and a Kruskal-Wallis test (P<0.05) was used to determine potential differences between groups. No differences were found between the six hours of the day in the velocity or the time active in the control groups on the stress (χ² = 4.829, d.f. = 5, P = 0.56 and χ² = 5.002, d.f. = 5, P = 0.74, respectively), alarm pheromone (χ² = 8.215, d.f. = 5, P = 0.33 and χ² = 2.155, d.f. = 5, P = 0.81, respectively) and caffeine challenges (χ² = 11.017, d.f. = 5, P = 0.14 and χ² = 12.735, d.f. = 5, P = 0.19, respectively). A Wilcoxon signed-rank test (P<0.05) was performed to assess any potential difference between the pre- (baseline) and post-stimulation average velocity and amount of time spent active on the same larvae in every Experiment. The change in average velocity and activity from pre-stimulation to post-stimulation states in each challenge was determined using a Kruskal-Wallis test (P<0.05). When Kruskal-Wallis test revealed any significant difference, post hoc Mann-Whitney U comparisons were made to compare each treatment group to both the control group using a Bonferroni correction, resulting in a significance level set at P<0.0031 (0.05/16).

## Results

### Impact of treatment on normal behavior

**Fig 2:**
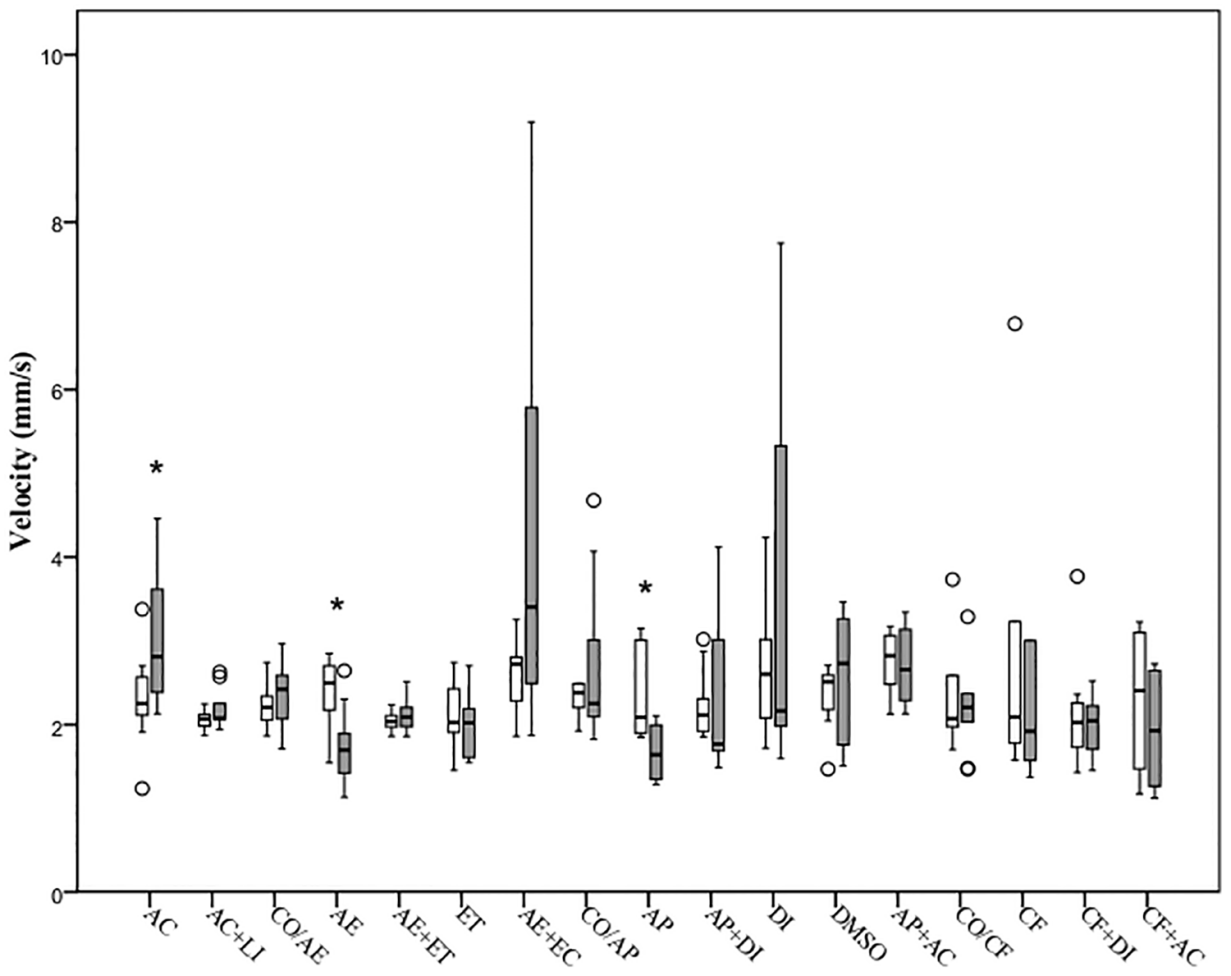
Velocity shown by 5dpf zebrafish during a 10-minute period before (white boxes) and after (grey boxes) exposure to different treatments. Each box shows the lower and upper quartile values and the central horizontal black line indicates the median value. The error bars indicate the variation for the rest of the data and outliers are indicated as white dots. Significant differences between pre- and post-stimulation behaviour are indicated with an asterisk (Wilcoxon signed-rank test, P<0.05; n = 10 per group).

**Fig 3:**
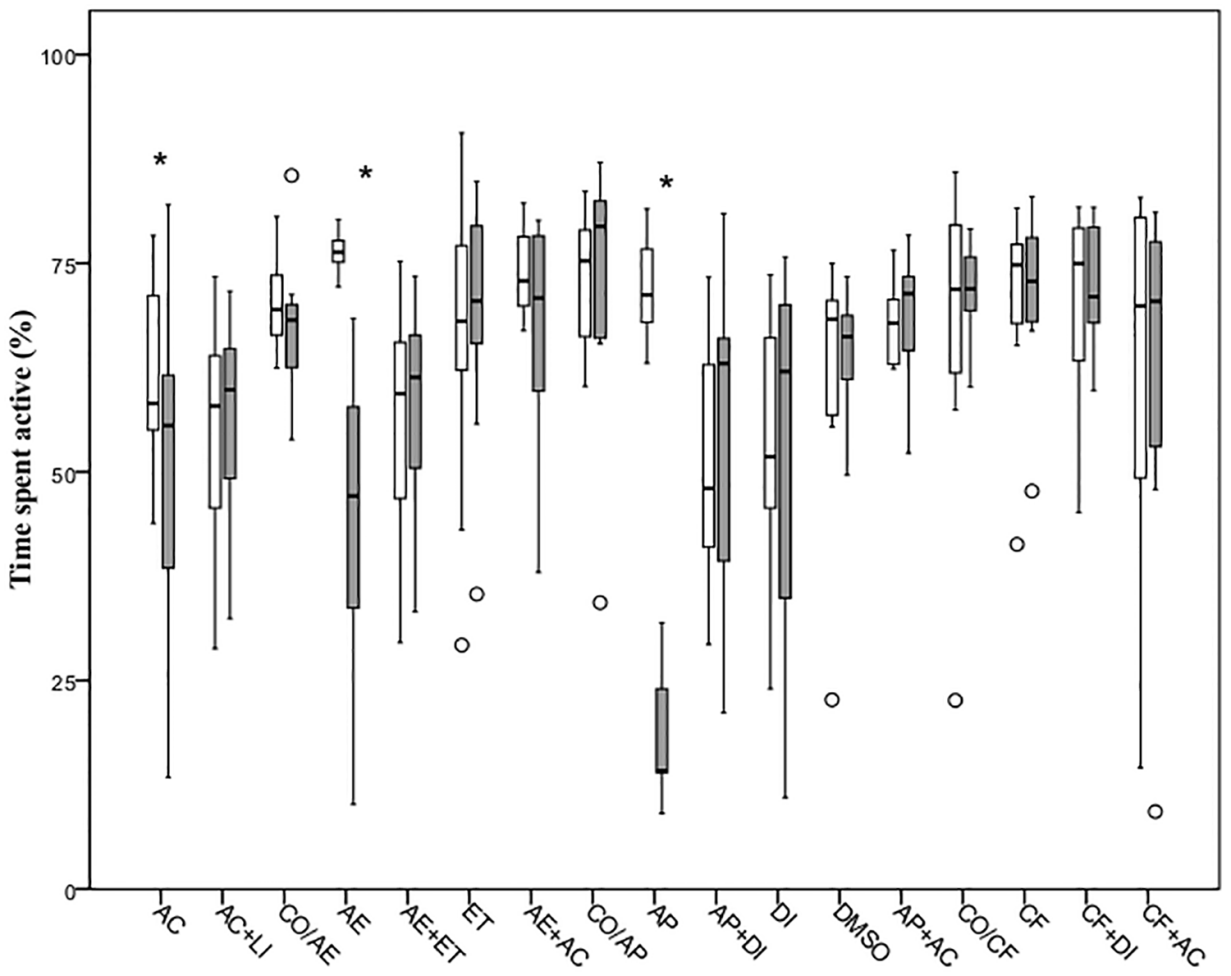
Time spent active shown by 5dpf zebrafish during a 10-minute period before (white boxes) and after (grey boxes) exposure to different treatments. Each box shows the lower and upper quartile values and the central horizontal black line indicates the median value. The error bars indicate the variation for the rest of the data and outliers are indicated as white dots. Significant differences between pre- and post-stimulation behaviour are indicated with an asterisk (Wilcoxon signed-rank test, P<0.05; n = 10 per group).

When comparing pre-treatment values with post-treatment within each group, exposure to acetic acid led to an increase in the swimming velocity (mm s^-1^) and a reduction in the general activity (%) (Figs 2 and 3). However, administration of lidocaine 5 mg l^-1^ ameliorated this effect, with fish in this group showing no differences in the parameters above mentioned. The air emersion challenge (AE) evoked a decrease in the swimming speed and the time spent active compared with the baseline values (Table 3). However, prior exposure to etomidate 0.5 mg l^-1^ did not alter this response to air emersion. Fish exposed to etomidate alone showed no significant change in any of the two indicators measured. Air emersion followed by exposure to acetic acid 0.1% did not influence swimming velocity or time spent active from pre-treatment values (Figs 2 and 3). No change in velocity nor time spent active was observed in the control group (CO/AE).

**Table 3:** Wilcoxon signed-rank test analysis of the velocity and time spent active in for each group before and after treatment. Significant results in bold.

	Velocity		Time spent active	
Group	Z	P	Z	P
AC	-2.50	**<0.001**	-1.48	**0.0014**
AC+LI	-0.66	0.51	-0.15	0.88
CO/AE	-0.764	0.44	-0.968	0.33
AE	-2.09	**0.037**	-2.80	**0.005**
AE+ET	-0051	0.96	-0.15	0.88
ET	-0.76	0.45	-0.56	0.58
AE+AC	-1.58	0.11	-1.17	0.24
CO/AP	-0.36	0.72	-0.46	0.65
AP	-2.29	**0.022**	-2.80	**0.005**
AP+DI	-0.26	0.80	-0.15	0.88
DI	-0.051	0.96	-0.050	0.95
DMSO	-0.36	0.72	-0.26	0.80
AP+AC	-0.051	0.90	-0.044	0.96
CO/CF	-0.051	0.97	-0.153	0.88
CF	-0.97	0.33	-0.15	0.88
CF+DI	-0.153	0.87	-0.26	0.80
CF+AC	-0.76	0.44	-0.26	0.80

Exposure to alarm pheromone for 10 minutes decreased both the time that the fish spent swimming and the swimming velocity, however animals treated with diazepam 5 mg l^-1^ prior to exposure to the alarm substance did not exhibit a change in behavior after alarm substance (Figs 2 and 3). Administration of diazepam and DMSO alone did not influence the behaviour of the larvae (Table 3).

Fish did not display any change in the swimming speed or in the proportion of time active when exposed to caffeine. Similarly, the group of animals exposed to the diazepam 10 minutes before the challenge with caffeine showed no differences in the parameters measured. No changes in the velocity or time spent active were observed in larvae exposed to the DMSO or diazepam (Table 3). Immersion in the acetic acid after exposure to caffeine (CF+AC) did not evoke any behavioural change relative to the baseline (pre-stimulation) state.

### Comparison between treatments

**Fig 4:**
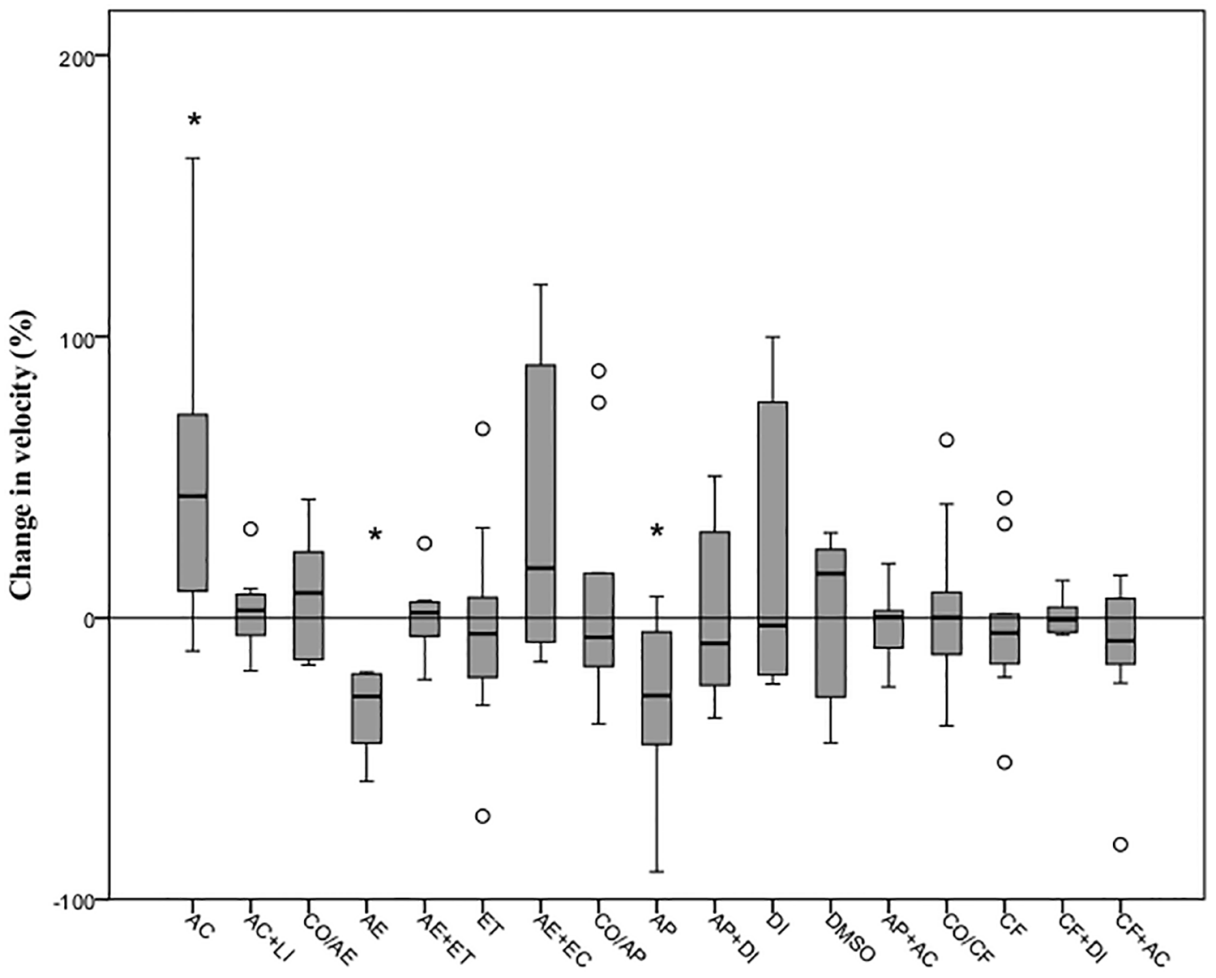
Change in velocity (%) shown by 5dpf zebrafish exposed to different treatments. Each box shows the lower and upper quartile values and the central horizontal black line indicates the median value. The error bars indicate the variation for the rest of the data and outliers are indicated as white dots. Significant differences between groups and the control groups are indicated with an asterisk (control in the air emersion challenge: CO/AE), a hashtag (control in the alarm pheromone challenge: CO/AP) or a cross (control in the caffeine challenge: CO/CF) (Wilcoxon signed-rank test, P<0.0031).

**Fig 5:**
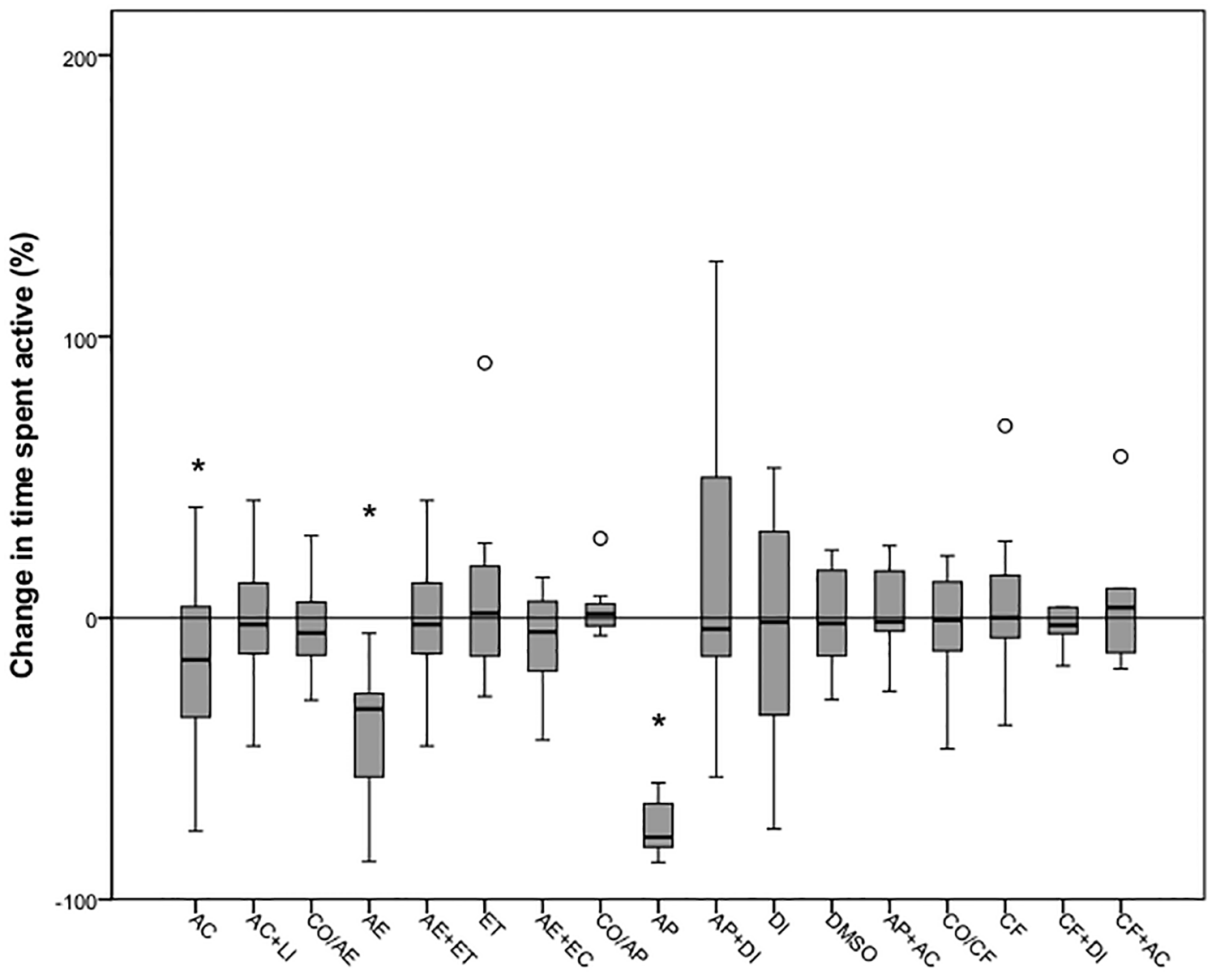
Change in time spent active (%) shown by 5dpf zebrafish exposed to different treatments. Each box shows the lower and upper quartile values and the central horizontal black line indicates the median value. The error bars indicate the variation for the rest of the data and outliers are indicated as white dots. Significant differences between groups and the control groups are indicated with an asterisk (control in the air emersion challenge: CO/AE), a hashtag (control in the alarm pheromone challenge: CO/AP) or a cross (control in the caffeine challenge: CO/CF) (Wilcoxon signed-rank test, P<0.0031).

There was a significant difference between all groups in the percentage change in swimming velocity (χ² = 37.41, d.f. = 16, P = 0.002) and in the time spent active (χ² = 43.62, d.f. = 16, P<0.001). Fish exposed to acetic acid 0.1% showed a significant increase in the swimming speed (U = 22, P<0.001) and a reduction in the time active (U = 38, P<0.001) relative to the control group (CO/AE). However, no changes were observed with exposure to lidocaine 5 mg l^-1^ prior to administration of acetic acid (U = 42, P = 0.58 and U = 44, P = 0.68, respectively). The air emersion challenge significantly decreased both the swimming speed and the time spent active compared to control larvae (U = 10, P = 0.002 and U = 10, P = 0.002, respectively), whereas fish exposed to 0.5 mg l^-1^ of etomidate prior to the challenge (AE+ET) showed no variation in any of the parameters (U = 39, P = 0.44 and U = 44, P = 0.68, respectively). The group administered with etomidate only (ET) showed no significant change in neither velocity nor the time active (U = 37, P = 0.35 and U = 41, P = 0.85, respectively) relative to the control group (CO/ET; Figs 4 and 5). Air emersion prior to exposure to the acetic acid prevented the decrease in swimming speed and time active observed in fish undergoing the air immersion challenge only (U = 34, P = 0.25 and U = 47, P = 0.85, respectively).

With regard to the alarm pheromone challenge, larvae exposed to acetic acid swam faster but for less time (U = 25, P<0.001 and U = 30, P<0.001, respectively) than those left undisturbed (CO/AP), whereas treatment with lidocaine (U = 38, P = 0.39 and U = 47, P = 0.85) prevented any changes relative to controls after exposure to the acid (Figs 4 and 5). The alarm pheromone evoked a significant decrease in velocity and time active (U = 26, P = 0.009 and U = 1, P<0.001, respectively) but fish exposed to diazepam 5mg l^-1^ 10 minutes prior to the alarm substance had a similar median velocity and time spent active (U = 49, P = 0.97 and U = 47, P = 0.85) compared with controls (CO/AP). Both the group treated with the organic solvent DMSO (U = 40, P = 0.48 and U = 44, P = 0.68) and the diazepam+DMSO (U = 44, P = 0.68 and U = 50, P = 0.99) did not show a significant change in the two parameters measured. The differences in behaviour observed after exposure to the alarm substance were not evident after immersion in acetic acid 0.1%, with similar median velocity and time spent active (U = 39, P = 0.44 and U = 46, P = 0.80, respectively).

Statistical analysis revealed that exposure to acetic acid exerted an increase in the swimming velocity and a reduction in the time spent active (U = 18, P00.001 and U = 35, P = 0.001, respectively) relative to controls (CO/CF). These changes in the control group were not observed when compared with larvae administered with 5mg l^-1^ of lidocaine after immersion in the acid (U = 43, P = 0.63 and U = 49, P = 0.97). When larvae were exposed to 100 mg l^-1^ of caffeine, no significant reduction in the time median velocity or time spent active were observed (U = 42, P = 0.58 and U = 45, P = 0.74, respectively). Immersion in diazepam 5 mg l^-1^ before exposure to caffeine had no significant effect compared with the control group (U = 49, P = 0.96 and U = 49, P = 0.97, respectively). Fish treated with both DMSO (U = 41, P = 0.53 and U = 49, P = 0.97) and diazepam+DMSO only (U = 49, P = 0.97 and U = 49, P = 0.95) displayed similar velocities and times active. The behavioural change observed in the caffeine-exposed fish was not apparent in those exposed to acetic acid 0.1% too, with similar median velocity (U = 43, P = 0.63), while there were no differences in the time active (U = 48, P = 0.91).

## Discussion

The present study has shown for the first time that larval zebrafish are significantly affected by stress, fear, and anxiety treatments that modulate the response to a noxious stimulus. Exposure to acetic acid for 10 minutes elicited significant behavioural changes in 5dpf zebrafish, with fish showing higher median swimming velocity (mm/s) and lower median time spent active (%) compared with the control group. Fish exposed to air emersion and diazepam swam slower and for less time, whereas those administered with caffeine only had a lower median velocity. Animals exposed to air emersion, diazepam and caffeine prior to the acetic acid challenge did not display these changes.

Larvae exposed to a solution of 0.1% of acetic acid showed a significant behavioural response from the baseline values (pre-stimulation), characterised by higher swimming velocity and a reduction in the time spent active. Although a reduction in the activity has been previously observed in larval zebrafish in our laboratory [35] and in fish exposed to potentially noxious stimulation [5,43,44], the increased swimming velocity has not been described in fish yet. Administration of lidocaine 5 mg/l prevented the behavioural changes observed. This local anaesthetic was first tested in fish in rainbow trout [45] and previous research confirmed its efficacy as anaesthetic in adult zebrafish [46]. Moreover, its use as analgesic in larval zebrafish exposed to acetic acid has been tested [36]. These results may imply that larvae experiences the stimulus as noxious.

Short-term air exposure has been demonstrated to increase cortisol levels in fish [47,48] and specifically in adult zebrafish [49] but no studies have identified the effects on larval zebrafish. In our study, larvae exposed to the air emersion challenge displayed a significant response, with reduced swimming velocity and general activity. Etomidate is an anaesthetic agent, analog of the metomidate, that affects adrenal steroidogenesis inhibiting production of cortisol and that has been used in fish [41], Administration of etomidate prevented the reduction in both velocity and locomotor activity. The group exposed to etomidate did not show any apparent behavioural response, which is in agreement with other studies [50]. Therefore, we hypothesize that etomidate was effective counteracting the effects of the air emersion and therefore, reducing the stress response of the larvae after this challenge. Only one study has evaluated the efficacy of etomidate or metomidate as an anaesthetic in larval fish [51]. These authors reported that metomidate was ineffective for red drum (*Sciaenops ocellatus*) and goldfish (*Carassius auratus*) larvae, although this conclusion is based on survival and recovery times and not on physiological indicators such as cortisol.

The alarm reaction consists of a set of behaviors that may protect fish from nearby active predators. This substance is synthesized in the epidermal cells of the fish and then released if there is cell membrane damage, which induces a strong fear response in nearby individuals [29,52]. Whereas new models for the study of fear and anxiety have been developed in larval zebrafish [30,31], only one study has determined the impact of the alarm substance in young stages of this species [53]. In this study, embryos exposed for 30 minutes displayed higher overall developmental rates compared with non-exposed fish. However, the effects on the behaviour of early developmental stages is unknown yet. This behavioural response can result in either increased [19] or reduced activity [20] in adult fish. In our study, larvae exposed to 3.5 ml/l of alarm pheromone, a concentration that has been successfully tested in adult zebrafish [21], presented similar responses to those exposed to the air emersion challenge, i.e. a decrease in the velocity and time spent active. Although this reduction in the general activity could indicate that larvae showed some kind of predator avoidance strategy, such as freezing, with our data it is not possible to discern whether fish actually detected the alarm pheromone. Further studies should attempt to detect characteristic abnormal behaviours in larval zebrafish such as erratic movements, wall-hugging or freezing [54]. Diazepam is an agent belonging to the group of benzodiazepines, which are widely prescribed for the treatment of anxiety and other disorders. In fish, it has been proved to reduce the responses elicited by fear or anxiety-like situations [39,40]. In our study, the group of larvae exposed to diazepam only did not show the behavioural changes observed in the group exposed to the alarm pheromone. Thus, we can conclude that this substance did not influence the behavioural responses of the larvae, which is consistent with previous research [31]. The results of the present study could imply that these fish actually underwent some kind of fear and/or anxiety when exposed to alarm substance and that this could be ameliorated using an anxiolytic substance, diazepam.

Caffeine acts as a stimulant at low doses and there is ample evidence that it elicits anxiety-like behaviours in zebrafish [29–31] at similar concentrations as the used in our experiment. Thus, it may seem prudent to expect an increase in both the swimming speed and time spent active in fish exposed to this substance. However, fish exposed to caffeine did not show any change in any of these indicators which is in agreement with previous studies on the behavioural responses of zebrafish to caffeine [31,55,56] but now with Richendrfer et al. [30], who found a reduction in the swimming speed with larval zebrafish. Thus, with our data it is not possible to conclude that the caffeine evoked an anxiety-like behavioural response. It may be that the concentration used in this experiment was too low to exert a significant change or that, caffeine exerted a different behaviour that was not quantified here. Therefore, further research is needed to fully understand the responses of larval fish to this substance.

Our results show that the behavioural responses observed in the group exposed to a potentially noxious stimulus, i.e. immersion in acetic acid 0.1%, is inhibited by previous exposure to either air emersion and alarm substance. These results may suggest an antinociceptive effect of stress, and fear or a mechanism that inhibit nociception in situations of stress and fear, which results in stress-induced analgesia. This phenomenon has been described in mammals [16,57,58] and fish [19] but never in larval fish. Stress induced analgesia refers to an animal not showing any signs of pain when exposed to a potentially painful stimulus after undergoing a stressful situation. This response is also present when fish are exposed to a fear- or anxiety-eliciting challenge. For some authors, fear and anxiety are undistinguishable, whereas others believe that they are separate mechanisms. Anxiety is a generalized response to an unknown threat or internal conflict, whereas fear is focused on known external danger [33]. Both phenomena are extremely difficult states to assess because they are a subjective experience. However, some investigators have explored the quantification of these defense reactions in fish [59,60]. These reactions are not independent of pain but are closely related [27,34]. It is known that fear and anxiety trigger the endorphin mechanism, therefore inhibiting pain motivation and recuperative behaviors that might compete with effective defensive behavior. A decrease in nociceptive sensitivity allows a threatened or injured animal to engage in necessary defensive behaviors, such as freezing, fleeing or fighting, by minimizing signals that would otherwise alert the animal to attend to an injury [22]. Evidence demonstrated the existence of an endogenous opioid system in zebrafish that is similar to those found in mammals [61] and an endogenous opioid antinociceptive system, activated by an acute stress, has been found in fish [19,20,23]. The molecular mechanisms of fear-conditioned and stress-induced analgesia in fish are not as well elucidated as they are in mammals. It seems that there are several systems that play a significant role in the modulation of the descending inhibitory pain pathway involving stress and fear-induced analgesia in mammals including monoaminergic [62], endocannabinoid [63,64], and opioid [65], as well as a GABAergic and glutamatergic signalling in specific brain regions [12]. Stress induced analgesia has been recorded in another fish species, the piaçu, where alarm substance and other stressor resulted in reduced nociceptive or pain-related behaviours [20,23]. Additionally in trout, high stress linked to low dominance status meant these fish showed little response to painful treatment (Ashley et al 2009). Future study of these mechanisms may clarify the underpinning molecular and physiological changes that accompany these behavioural responses.

We hypothesize that an antinociceptive mechanism elicited by stress, fear and anxiety could have prevented the larvae to display the characteristic behavioural response caused by the noxious acetic acid.

## Conclusions

The novel results presented in this study demonstrate that 5dpf zebrafish showed altered behavioural responses after exposure to a potentially noxious stimulus and that these responses were inhibited by stress- and fear-eliciting situations. Therefore, this suggests the presence of a modulation mechanism of nociception in larval zebrafish, which is activated under potentially threatening or aversive situations. We believe that these findings are also relevant to welfare and handling in experiments involving the use of fish. Since these unprotected 5dpf larval zebrafish display similar behavioural responses to both noxious treatment and the various drugs tested in the present study, they could be proposed as a suitable alternative to replace adult zebrafish in nociception and analgesic studies. These results may propose the question as to whether 5dpf zebrafish should be protected if they show similar responses to potentially painful stimuli, however, further evidence is required such as brain processing mechanisms, altered future motivational state and an ability to learn to avoid noxious stimuli (see Sneddon et al. 2014) before this question can be fully answered.
